# Diagnostic Biomarkers of Periodontitis and Peri‐Implantitis: The Past, the Present, the Future

**DOI:** 10.1111/jre.70140

**Published:** 2026-07-19

**Authors:** Denis F. Kinane, Başak Bıyıkoğlu

**Affiliations:** ^1^ Periodontal Department University of Bern Bern Switzerland; ^2^ Department of Periodontology, Faculty of Dentistry Istanbul University‐Cerrahpaşa Istanbul Turkey

**Keywords:** biomarkers, gingival crevicular fluid, peri‐implant crevicular fluid, peri‐implantitis, periodontal diagnostics, periodontitis

## Abstract

In this narrative review we present our perspective on the past, present and future of periodontal and peri‐implantitis diagnostic biomarkers. This topic parallels aetio‐pathogenic research in that our understanding of the pathogenic, biological and biochemical mechanisms of these diseases is deeply entwined in the search for potential biomarkers of utility to diagnose them. Germane to this point is the marked success of bone‐related molecules in saliva compared to cytokines and metalloproteinases in differentiating soft and hard tissue destruction. Despite these advances, translation into routine practice remains limited. Methodological heterogeneity, lack of standardised sampling protocols, biological variability, and insufficient longitudinal validation continue to impede progress. The roadmap ahead requires harmonised protocols, robust multicentre studies, clinically validated thresholds, and technologies that are rapid, affordable, and easily integrated into daily workflows. Biomarkers combined with other factors and interpreted by AI tools may be utilised in future to disclose current disease, predict future disease and aid in monitoring treated conditions: they may provide prognostic tools and have potential in epidemiological screening, particularly if readily available and easily accessible sample matrices such as saliva are used. Ultimately, the future of periodontal and peri‐implant diagnostics lies in biologically informed, multi‐modal assessment frameworks that complement traditional examination rather than replace it. As molecular technologies mature and AI‐assisted personalised models evolve, biomarker‐based diagnostics have the potential to transform early detection, risk prediction, and treatment monitoring—bringing us closer to precision periodontology and implantology. The salivary sample matrix is feasible for home use with utility for population screening, whereas the gingival and peri‐implant crevicular fluid and the simple periodontal probe will continue to be invaluable in the analysis of localised disease.

## Graphical abstract

Diagnostic biomarkers of periodontitis and peri‐implantititis from the past, through the present, and into the future.

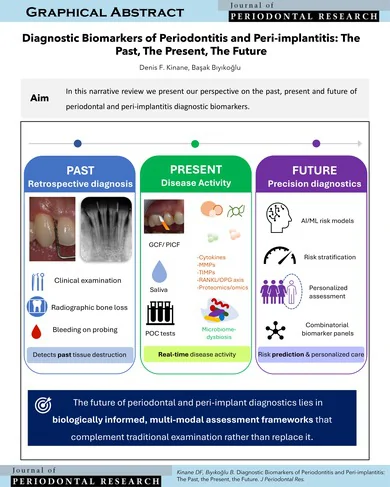

## 1 Introduction

Periodontitis is a multifactorial chronic inflammatory disease characterised by the loss of attachment apparatus and the supporting structures of the teeth. It is one of the most prevalent human chronic diseases, and progresses without pain or early symptoms, resulting in delayed patient presentation and typically a diagnosis at an advanced stage [1, 2, 3]. Once the irreversible alveolar bone loss occurs, it is challenging for the clinician to prevent further tooth loss despite multiple advances in periodontics. Dental implants have emerged over the last few decades to replace lost teeth and in doing so present us with new challenges in their maintenance. At the outset, peri‐implantitis was not widely foreseen, and thus the similarities and differences between periodontitis and peri‐implantitis aetiology, diagnosis and treatment had to be learned and acted upon quickly. Peri‐implantitis, as the name suggests, is a chronic inflammatory pathological condition resulting in the destruction of peri‐implant tissues [4]. Both periodontitis and peri‐implantitis are initiated by bacterial challenge, and subsequent hard and soft tissue destruction occurs through the interplay of biofilm bacteria and the host response. Although the clinical presentation of these two conditions is similar, once tissue destruction is initiated, the rate of bone loss around implants is faster and more difficult to manage compared to that around teeth [5]. The fundamental difference between implants and teeth, apart from the titanium and calcified human tissue aspect, is that implants have no periodontal ligament and thus a major difference in blood supply to affected areas is seen when compared to natural teeth: this lack of vascularity and ability to recruit both soluble and cellular defensive elements, more than anything, may explain the differences in destructive response seen. Moreover, titanium particles (TPs) seen in the peri‐implant tissues may trigger an inflammatory response that may also account for the non‐linear bone‐loss pattern in peri‐implantitis [6, 7, 8]. Both entities are related to poor quality of life and not only constitute a public health issue but also an economic burden [9, 10, 11]. Therefore, detection of patients and sites with these conditions, and at higher risk of these conditions, is essential to prevent irreversible consequences.

## 2 Past: Conventional, Retrospective Diagnostic Paradigms

Periodontal diagnostics have historically been based on “clinical and radiographic parameters” that document the cumulative effects of disease. Measurements such as probing pocket depth (PD), clinical attachment level (CAL), bleeding on probing (BOP), tooth mobility, and radiographic assessment of alveolar bone loss have long served as the foundation for diagnosing and classifying periodontal diseases [10]. These parameters remain essential for determining disease severity and extent; however, they primarily reflect “past periodontal destruction” rather than current disease activity. Like periodontitis, the diagnosis of peri‐implantitis is mainly based on clinical and radiographic examination. According to the consensus report of the World Workshop on the Classification of Periodontal and Peri‐implant Diseases and Conditions, the diagnosis of peri‐implantitis requires the evaluation of bleeding and/or suppuration on probing, probing depth, and bone loss [4]. Further to this Classification World Workshop, a recent European Workshop in Periodontology has focused on diagnostics, and many of the issues raised by these experts will be included in this briefer narrative review [12].

Clinical parameters are further limited by “measurement variability”, which may be influenced by probe design, examiner technique, inflammatory status of the tissues, and site‐specific anatomical factors [10, 13]. Importantly, while clinical and radiographic assessments provide a snapshot of periodontal and/or peri‐implant status at a given time point, they offer limited insight into “future risk of progression”, particularly in patients with similar clinical presentations but differing biological susceptibility [13, 14]. It should be kept in mind that the cyclical character of periodontal destruction is another factor that limits the accuracy and predictability of clinical examination [15]. The presence of bleeding on probing is widely accepted as a sensitive and accurate indicator of gingival inflammation; however, the classic study by Lang revealed that its positive predictive value for future disease progression remains low (6%). In contrast, the absence of BOP has a high negative predictive value (98%), supporting its reliability as a marker of effective periodontal maintenance [16]. Furthermore, BOP does not necessarily capture the complex host–microbial interactions underlying active tissue breakdown. Consequently, while BOP retains clinical relevance, particularly during supportive therapy, it should be interpreted alongside other clinical parameters and emerging biomarkers to achieve a more comprehensive and precise assessment of periodontal disease activity.

As the understanding of periodontal and peri‐implant pathogenesis evolved, it became evident that disease initiation and progression result from a complex interaction between “microbial dysbiosis and host immune‐inflammatory responses”, modulated by genetic, systemic, and environmental factors [17, 18, 19]. Despite this growing biological insight, diagnostic approaches remained largely unchanged, leaving clinicians without reliable tools to identify “active disease phases”, differentiate “progressive versus stable sites”, or predict disease trajectory in individual patients [13, 20]. Although still considered the gold standard, comprehensive periodontal examination is time consuming, requires experience and has its own limitations such that there is opportunity for biomarkers to help overcome these limitations [21].

The definition of a biomarker is “a defined characteristic that is measured as an indicator of normal biological processes, pathogenic processes, or responses to an exposure or intervention, including therapeutic interventions” [22]. Periodontal biomarkers can be classified by integrating their biological origin with their clinical diagnostic applicability, thereby reflecting the multifactorial nature of periodontal disease. Microbial biomarkers primarily indicate the presence of periodontal pathogens or their virulence factors and therefore can be considered biomarkers of exposure [21, 23, 24]. Meanwhile, host‐derived inflammatory mediators provide insight into the current inflammatory burden and may serve both diagnostic and prognostic purposes [23].

Biomarkers associated with tissue degradation and alveolar bone metabolism are more directly linked to ongoing periodontal destruction and may offer greater prognostic relevance [21, 25]. Additionally, regulatory and anti‐inflammatory mediators may help predict treatment response by reflecting the host's capacity to modulate inflammation. Genetic and epigenetic markers further contribute to risk assessment by identifying susceptible individuals, however, as classical non‐modifiable risk factors, their clinical translation remains limited [21, 22].

In medicine, evaluation of the biomolecules in different biological fluids for diagnostic purposes has been performed historically [26]. Oral fluids such as saliva, gingival crevicular fluid (GCF), and peri‐implant crevicular fluid (PICF) around dental implants offer site‐specific, non‐invasive opportunities to monitor a wide array of microbial and host‐derived biomarker candidates for diagnostic, prognostic and risk assessment purposes. Although not site‐specific and comparatively invasive, serum is also a source of possible biomarkers. (Figure 1).

**FIGURE 1:**
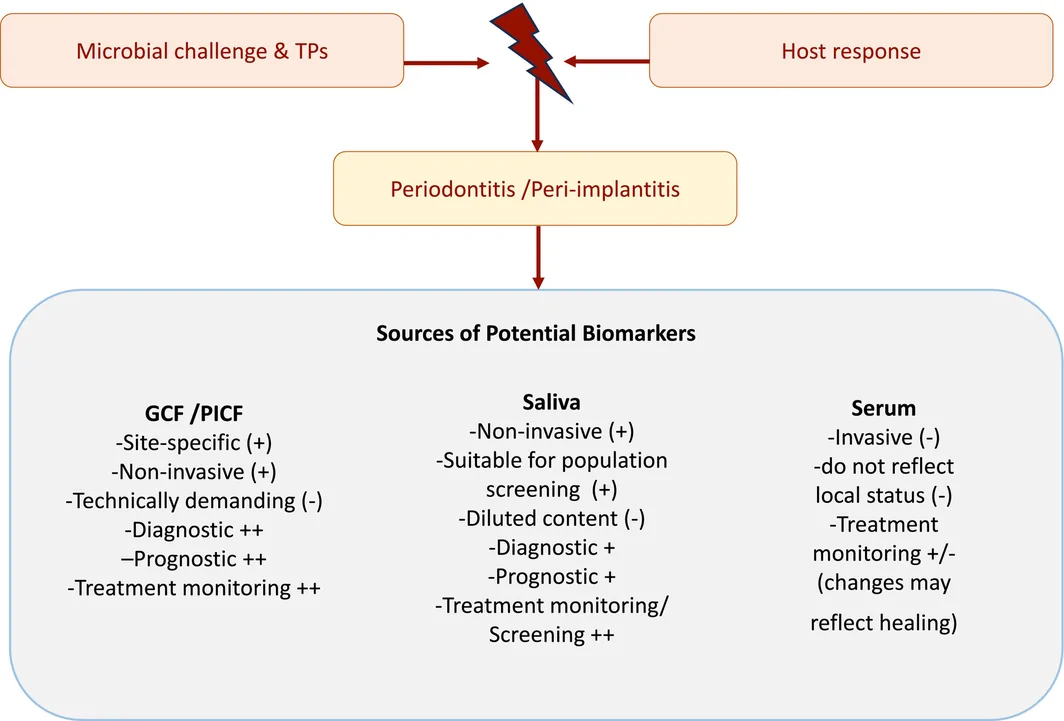
Conceptual framework linking etiopathogenesis of periodontitis and peri‐implantitis to biomarker sources and their expected clinical applications.

Salivary biomolecules have been shown to correlate with oral diseases including periodontitis. Besides, saliva holds many advantages as it can easily be collected by even lay persons non‐invasively [26, 27]. Saliva is derived from serum and salivary gland secretions. The limitation of saliva as a source of biomarkers is that its constituents are generally, rather than locally produced, and include GCF and bacteria from multiple sites as well as oral epithelial cells and foreign substances such as food. Also, the large aqueous component may dilute other components and make them less detectable [27]. GCF, on the other hand, is a localised serum exudate/transudate and its increased volume and constituent concentrations provide indicators of local inflammation [28, 29, 30].

As the history of peri‐implant diseases is briefer than periodontitis, the utility of PICF for diagnostic and prognostic purposes is rather new. The first researchers to show the presence of fluid flow that is comparable to GCF were Apse et al. in 1989 [31]. In their study, the researchers showed the presence of collagenases and their inhibitors in PICF [31]. At this point in this narrative review, and consistent with other articles in this series, we wish to present a historical perspective of one of the co‐authors, and consistent with the series this will be provided as written in the first person.

### 2.1 A Past Perspective

My early fascination with periodontology came from viewing the gingival crevice as a war zone where host cells confronted anaerobic bacteria. Discovering that this battle extended into the gingival crevicular fluid (GCF)—and that this fluid could be collected, viewed and analysed—was compelling. Using dark‐field microscopy to show patients their subgingival plaque was never diagnostic, but it was an effective and memorable motivator.

With a PhD in immunology and microbiology from Edinburgh, I was naturally drawn to the diagnostic potential of GCF. The pre‐1950s literature of Brill and Björn established GCF as an inflammation‐related transudate rather than saliva, though their focus was largely physiological [ 28 ]. Giorgio Cimasoni's encouragement and monographs further stimulated interest in GCF as a diagnostic medium, beginning with simple biomarkers such as albumin and immunoglobulins [ 32 ]. The work progressed and additional molecules such as cathepsins, and elastases and other host‐derived molecules were reported on [ 33 ] .

My own research centred on neutrophils, monocytes, and immunoglobulins in periodontal disease, including disproving the proposed neutrophil chemotaxis defect in juvenile periodontitis [ 34 ] .

During sabbaticals in Bern and visits to Geneva, we characterised protease inhibitors, enzymes, and immunoglobulins within the crevice [ 35 ] , recognising that GCF contained both systemic transudate and local inflammatory exudate. These enzymes and inhibitors reflected the host‐driven tissue destruction that occurs as a controlled retreat from microbial challenge embedded in calculus.

*Attempts to distinguish active from inactive disease using biomarkers—and to use immunoglobulins as indicators of activity—proved far more difficult than hoped* [ 36 ]. It became increasingly clear that, despite the scientific promise of complex molecular assays, none could match the speed, simplicity, and clinical utility of the periodontal probe for detecting calculus, bleeding on probing, pocket depth, and attachment loss in real time.

## 3 Present: Biomarker‐Based and Biologically Informed Diagnostics

Contemporary periodontal diagnostics increasingly reflect the recognition of periodontitis and peri‐implantitis as a ‘multifactorial, host‐mediated inflammatory disease’ characterised by site specificity and episodic progression [17, 18]. This paradigm shift has driven substantial interest in the identification of ‘biological markers’ that reflect real‐time molecular events within periodontal/peri‐implant tissues and may complement traditional clinical assessment.

Among available biofluids, GCF has emerged as a particularly valuable diagnostic sample matrix. GCF is easily and non‐invasively collected, allows repeated sampling, and provides ‘site‐specific information’ that directly reflects the local host–microbial interaction [29, 37]. Its complex composition includes inflammatory cytokines, host‐derived and bacterial enzymes, oxidative stress markers, tissue breakdown products, antibodies, and bone metabolism regulators, making it highly relevant for periodontal diagnostics [37]. Peri‐implant crevicular fluid, on the other hand, is the equivalent of GCF in dental implants and in a similar manner acts as a source of biomolecules reflecting the inflammatory status of peri‐implant tissues [38]. Both fluids are of importance because they provide site‐specific information compared with saliva. Particularly, in peri‐implant versus periodontitis comparisons, it may transpire that given the lack of periodontal ligament around implants and thus the reduced vascularity adjacent to the implant lesions, that PICF will be more disease exudate than vascular transudate, compared to periodontal site GCF.

Over the past five decades, numerous components of saliva and GCF have been evaluated as candidate biomarkers of periodontal disease activity and progression [29, 37, 39]. These biomarkers broadly correspond to key phases of periodontal pathogenesis: inflammation (e.g., interleukin (IL‐1β), IL‐6, TNF‐α), connective tissue degradation (e.g., matrix metalloproteinases), oxidative stress (e.g., malondialdehyde, antioxidant enzymes), and bone turnover (e.g., receptor activator of NF‐κβ ligand (RANKL), osteoprotegerin (OPG), C‐telopeptide pyridinoline cross‐links (ICTP)) [20, 29, 37].

Among these, ‘matrix metalloproteinase‐8 (MMP‐8)’—particularly its active form (aMMP‐8)—has been most consistently reported. MMP‐8 is a dominant collagenase in periodontal tissues and plays a central role in connective tissue destruction and repair processes [40, 41, 42]. Systematic reviews and meta‐analyses have shown that GCF MMP‐8 exhibits ‘good sensitivity and excellent specificity’ for diagnosing periodontitis, outperforming many other single biomarkers [43] and have also been associated with disease severity, microbial dysbiosis, and active tissue breakdown [44, 45, 46, 47, 48]. However, active or not, MMP‐8 is conceptually troublesome as a diagnostic biomarker due to the body's use of the metalloproteinases for all inflammation not just that which causes bone loss, and in tissue healing and repair. Thus, differentiating inflammatory states with and without bone loss and healing versus active repair sites is fraught. That is not to say this biomarker may not have utility, even non‐hypothesis‐driven biomarker candidates such as the micro‐RNAs can be useful diagnostically. One could reasonably argue that the critical diagnostic task of MMPs together with cytokines is in clinically differentiating ‘soft‐tissue‐only lesions’ (gingivitis and mucositis), from periodontitis and peri‐implantitis (where bone is lost). This may explain why bone destruction specific molecules such as RANKL and OPG show diagnostic accuracy (DA) superiority (AUC 0.91). In fact, combinations of metalloproteinases and cytokines had better DA in differentiating disease from health but not in periodontitis/gingivitis discrimination. Bone markers in saliva had the best DA (AUC 0.91) compared to cytokines (AUC 0.86) and metalloproteinases (AUC 0.77) [49].

Importantly, these findings have led to the development of **point‐of‐care and chairside diagnostic tests**, particularly lateral‐flow immunoassays capable of detecting aMMP‐8 in GCF, saliva, or mouthrinse samples [46, 47, 48]. These tests provide rapid, minimally invasive assessment of periodontal disease activity and show promise as adjuncts to conventional diagnostics. However, their clinical utility remains dependent on accurate validation against traditional clinical parameters, which themselves are retrospective in nature [50]. In addition, MMPs are only one of many molecules derived from both disease tissues and the vascular exudate and will be highly correlated with multiple cytokines, inflammatory markers, and transudate proteins, in the same way that simple albumin is. Thus, the uniqueness could be questioned, but not the enthusiasm with which its proponents promote it.

Beyond single biomarkers, recent systematic reviews emphasise that ‘combinations of biomarkers’—especially pro‐ and anti‐inflammatory cytokine pairs—may provide superior diagnostic accuracy, reflecting the complex biology of periodontal disease [51]. In parallel, ‘proteomic and metabolomic approaches’ have enabled large‐scale identification and quantification of GCF proteins and metabolites, revealing distinct molecular signatures associated with disease severity and progression [52, 53, 54, 55]. Despite encouraging findings, the translation of biomarker‐based diagnostics into routine clinical practice remains limited by methodological heterogeneity, lack of standardisation, and insufficient large‐scale longitudinal validation [29, 55].

Both peri‐implantitis and periodontitis have a microbial aetiology, yet the composition of the peri‐implant biofilm is different from that found in periodontitis. The difference is partly attributed to the interaction of the surface characteristics of titanium with the oral microenvironment [5]. Among periodontopathogens, *Treponema denticola* *(T. denticola)* was found to be consistently associated with peri‐implantitis, and its diagnostic accuracy is improved when combined with host response markers. The diagnostic accuracy for active peri‐implant disease was further enhanced when detection of *T. denticola* was combined with elevated PICF levels of IL‐1β, tissue inhibitors of metalloproteinase‐2 (TIMP‐2), and vascular endothelial growth factor (VEGF) compared with any single parameter alone [56]. VEGF was also evaluated for its capacity as a biomarker of peri‐implantitis [8] and its expression was significantly higher in peri‐implantitis granulation tissue than in periodontitis. Neovascular network density, which is characterised by hyperaemic vessels and zones of micro‐bleeding concentrated in contact zones toward the implant surface, was significantly higher in peri‐implantitis compared with periodontitis. This finding suggests a reactive angiogenic response to the intensive inflammatory environment at the biofilm‐contaminated implant surface. The authors also reported that TPs were present in all peri‐implantitis specimens; however, no histopathological pattern variation was observed around TPs. Soluble VEGF in PICF was positively correlated with clinical endpoints and demonstrated high specificity for peri‐implantitis (96%) [8].

Beyond microbial factors, the presence of titanium particles (TPs) released in the peri‐implant tissues may further contribute to the accelerated progression of peri‐implantitis compared with periodontitis. Although the initiation of peri‐implantitis is attributed to plaque accumulation, TPs and corrosion products have also been suggested to trigger a local aseptic inflammatory response [6, 7, 8]. The detrimental effect of TPs needs further investigation as titanium dioxide is widely used in sunscreens at considerably higher concentrations with minimal adverse effect attributed to them. The acidic microenvironment may reduce the resistance of titanium to corrosion which in turn upregulates M1 macrophage activity, promotes pro‐inflammatory cytokine release and stimulates osteoclastic differentiation via upregulation of RANKL [57].

Similar to periodontitis, aMMP‐8 has been studied extensively for its utility for peri‐implant diagnostics. Significantly elevated PICF aMMP‐8 levels were demonstrated across healthy implants (13.65 ± 7.18 ng/mL), peri‐implant mucositis (32.33 ± 21.20 ng/mL), and peri‐implantitis (73.07 ± 43.93 ng/mL) when the ImplantSafe point‐of‐care test was employed [58]. Interestingly, it was reported that an optimal cutoff of 33.7 ng/mL yielded 91.7% sensitivity and 75% specificity for identifying peri‐implantitis, outperforming bleeding on probing from the same site (sensitivity 83.3%, specificity 58%) [58]. Fragkioudakis et al. [59] confirmed these findings in an independent cohort, reporting sensitivity of 81.25% and specificity of 74.07% at a 20 ng/mL cutoff. However, longitudinal studies with larger sample sizes are needed to justify the employment of chair‐side aMMP‐8 tests in a clinical setting as well as a full analysis of this test's cost‐effectiveness and practical clinical utility.

The OPG/RANKL axis is significantly dysregulated in peri‐implantitis. In an earlier study, RANKL was shown to increase with disease severity and the OPG/RANKL ratio was highest in healthy implants while lowest in severe peri‐implantitis [60]. Rakic et al. [61] demonstrated that RANKL and OPG concentrations in PICF are significantly elevated in both peri‐implant mucositis and peri‐implantitis compared with healthy implants, with 100% accuracy, sensitivity, and specificity of RANKL for the diagnosis of peri‐implant mucositis. Importantly, approximately 40% of mucositis cases demonstrated RANKL levels comparable to or exceeding those in peri‐implantitis—a finding interpreted as evidence that active osteoclastogenic activity may be already underway before the radiographic threshold for peri‐implantitis is crossed. In the same study, development of a personalised diagnostic model (PDM) for implant monitoring was investigated. The PDM, which integrates BOP, probing depth, and RANKL, showed an accuracy of 96.27%, sensitivity of 95%, and specificity of 100%, which substantially outperforms any clinical parameter alone. Consistent with these findings, Chaparro et al. [62] demonstrated that RANKL and APRIL were significantly elevated in peri‐implantitis compared with healthy and mucositis implants. A multivariate model combining PICF‐APRIL with plaque index score achieved an AUC‐ROC of 0.95, with 94.4% sensitivity and 83.3% specificity, reinforcing the value of biomarker‐clinical parameter combinations over single‐marker approaches.

Peri‐implantitis is defined histo‐pathologically but is not fully characterised at the molecular level. In recent years, much non‐hypothesis‐driven research has been conducted, and microRNAs (miRNAs), which play a crucial role in physiological and pathological processes are of interest for their possible role as disease biomarkers. In an experimental peri‐implantitis study in dogs, miRNAs, let‐7, miR‐27a, and miR145 in peri‐implant tissue samples were shown to increase [63]. In another clinical study, the expression of let‐7 in PICF increased 94‐fold, indicating its particular role in peri‐implantitis. It must be noted that the actual function of miRNAs is not fully understood and that these biomarkers must currently be considered more ‘associated’ than ‘causal’ in diagnosis. Also, next‐generation sequencing technologies have rendered identification of unculturable pathogens possible, and it was shown that disease severity was correlated with the shift toward dysbiosis [64].

The potential of the peri‐implant plaque microbiome as a diagnostic tool was evaluated using shotgun metagenomics and consistent associations between microbiome composition and different peri‐implant statuses were reported [65]. Peri‐implantitis was associated with a highly specific and convergent microbiome signature, which in turn resulted in highly accurate results obtained by machine‐learning models to differentiate peri‐implantitis from health with an AUC of 0.78–0.96. Also, in another study [66], combining full‐length 16S rRNA sequencing with metatranscriptomics, it was demonstrated that integrating taxonomic and functional microbial data further improved predictive accuracy to AUC = 0.85, and identified enzymatic biomarker candidates such as urocanate hydratase, tripeptide aminopeptidase, and NADH:ubiquinone reductase that distinguish peri‐implantitis at the functional level independent of microbial taxonomy. Amino acid catabolism pathways, particularly histidine, lysine, and tryptophan utilisation, were reported to be significantly upregulated in peri‐implantitis biofilms, producing cytotoxic end‐products that contribute to tissue destruction through mechanisms distinct from the classical host inflammatory response.

These findings highlight both the promise and the complexity of peri‐implantitis biomarker research. The distinct histopathological, immunological, and microbial profile of peri‐implantitis justifies the development of disease‐specific biomarker panels rather than direct translation of periodontitis markers to peri‐implantitis. The most promising approaches seem to be combining PICF host‐response markers (aMMP‐8, RANKL, APRIL, IL‐1β) with microbial profiling and clinical parameters in personalised models, rather than relying on any single biomarker. The potential of peri‐implantitis biomarker candidates may extend from diagnosis to treatment‐response monitoring. In a prospective clinical study, a significant reduction in submucosal pathogen levels and in PICF concentrations of IL‐1β, VEGF, and IL‐6 at six months following non‐surgical peri‐implantitis therapy was reported with a sensitivity of 77% for predicting a favourable treatment outcome [67]. This finding suggests that PICF biomarker profiles may serve not only as diagnostic indicators of disease activity but also as real‐time monitors of therapeutic response. Host susceptibility, inter‐individual variability in cytokine response, the absence of a periodontal ligament, and the presence of TPs represent biological confounders that must be addressed in future large‐scale prospective validation studies. The selection of biofluid to be collected, standardisation of PICF and/or saliva collection protocols, consensus on diagnostic thresholds, and adherence to biomarker validation guidelines remain the critical translational steps before any composite biomarker panel can be recommended for routine clinical use in peri‐implantitis.

## 4 Challenges to the Clinical Translation of Biomarkers

### 4.1 General Challenges

Despite significant advances in biomarker research in recent years, the translation of these findings into daily clinical practice has been limited. Numerous biomarkers associated with inflammation, tissue degradation, and bone metabolism have been identified; however, only a few have progressed from bench to chairside. To do so, four main steps were described previously [21]: (1) descriptive and mechanistic studies that define biomarker candidates and the role they play in disease; (2) validation of the power of a candidate biomarker in independent populations; (3) development of fast and reliable assays for detection of the biomarker; and (4) clinical validation of the aforementioned assay. On the successful completion of these four steps, we deliver a biomarker with potential clinical utility for periodontal and/or peri‐implant diagnostics, but this is a false summit. Cost‐effectiveness, true clinical utility of the diagnostic, ease of use, and a variety of difficult‐to‐predict parameters can determine whether this diagnostic tool succeeds. In terms of unpredictable parameters, an example of this is the VHS tape versus the technically superior Betamax race, where VHS won out due to the greater availability of VHS recording machines. The same may hold out in periodontics, where simplicity and availability might be more highly prized. Being first‐to‐market may offer a considerable advantage also and for this we need to bear in mind that many of the diagnostics discussed here are not fully validated and many are more conceptual than currently practical.

### 4.2 Specific Challenges

One of the primary problems is the lack of standardised sampling protocols. Different biological fluids, including saliva, gingival crevicular fluid (GCF), peri‐implant crevicular fluid (PICF), and serum, can be used for biomarker research. However, these fluids differ considerably in their composition and biomarker concentrations. Saliva offers advantages in terms of non‐invasive collection and ease of handling, but it contains diluted biomarker levels, and it also reflects serum and GCF components. In contrast, GCF and PICF provide site‐specific information but require more technically demanding collection procedures and yield smaller volumes of samples. Variations in sampling techniques, collection devices, and storage conditions can significantly influence biomarker measurements, limiting comparability across studies [21].

Another challenge is biological variability among individuals. Periodontal diseases are multifactorial conditions influenced by genetic predisposition, systemic health, environmental factors, and behavioural habits such as smoking and oral hygiene practices. Descriptive characteristics of participants may directly affect the study outcomes if there are biases in the participant recruitment [21]. The episodic or cyclical nature of periodontal disease progression may also be considered a limitation [15]. Therefore, longitudinal studies are required to safely determine the predictive value of specific biomarkers [25, 68].

As emphasised in recent reviews and meta‐analysis the identification of a single biomarker capable of accurately diagnosing periodontal disease is not realistic [29, 43, 69]. In order to overcome this limitation, combined biomarkers are recommended to be used in diagnostic panels that integrate host response, microbial profiles, and tissue degradation markers [43, 69].

There have been advances in biomarker detection methods in the last decades. The conventional laboratory techniques such as enzyme‐linked immunosorbent assays (ELISA), polymerase chain reaction (PCR), or mass spectrometry provide high analytical sensitivity and specificity; however, they are expensive, time‐consuming, and require training [70]. The development of rapid point‐of‐care diagnostic devices has therefore become a major focus of recent research. To date, rapid chairside tests have been developed for the detection of aMMP‐8 in saliva, GCF and PICF to diagnose periodontitis and peri‐implantitis [44, 46, 47, 48].

The existence of a particular biomolecule does not necessarily indicate its useful role as a diagnostic biomarker [71]. Diagnostic capability investigations require specific accuracy studies in order to estimate the sensitivity and specificity of the test. It is recommended to define a cut‐off value to obtain comparable threshold levels that can discriminate health and disease. The presentation of sensitivity and specificity values of a defined cut‐off value can provide clear insight on the effect of covariates on the diagnostic capability of the biomarker [21, 43].

In summary, although periodontal biomarker research has generated considerable knowledge regarding the molecular mechanisms of periodontal and peri‐implant diseases, several barriers continue to limit clinical translation. To overcome these limitations, Gursoy and Kantarci proposed a roadmap that with the intention of improving data quality, validation, reproducibility and making clinical translation possible [21]. The definition of health and disease, standardisation of sampling protocols, identification of reliable biomarker panels, development of cost‐effective point‐of‐care technologies, and validation through large longitudinal studies are essential steps toward the successful integration of biomarker diagnostics into precision periodontology (Figure 2).

**FIGURE 2:**
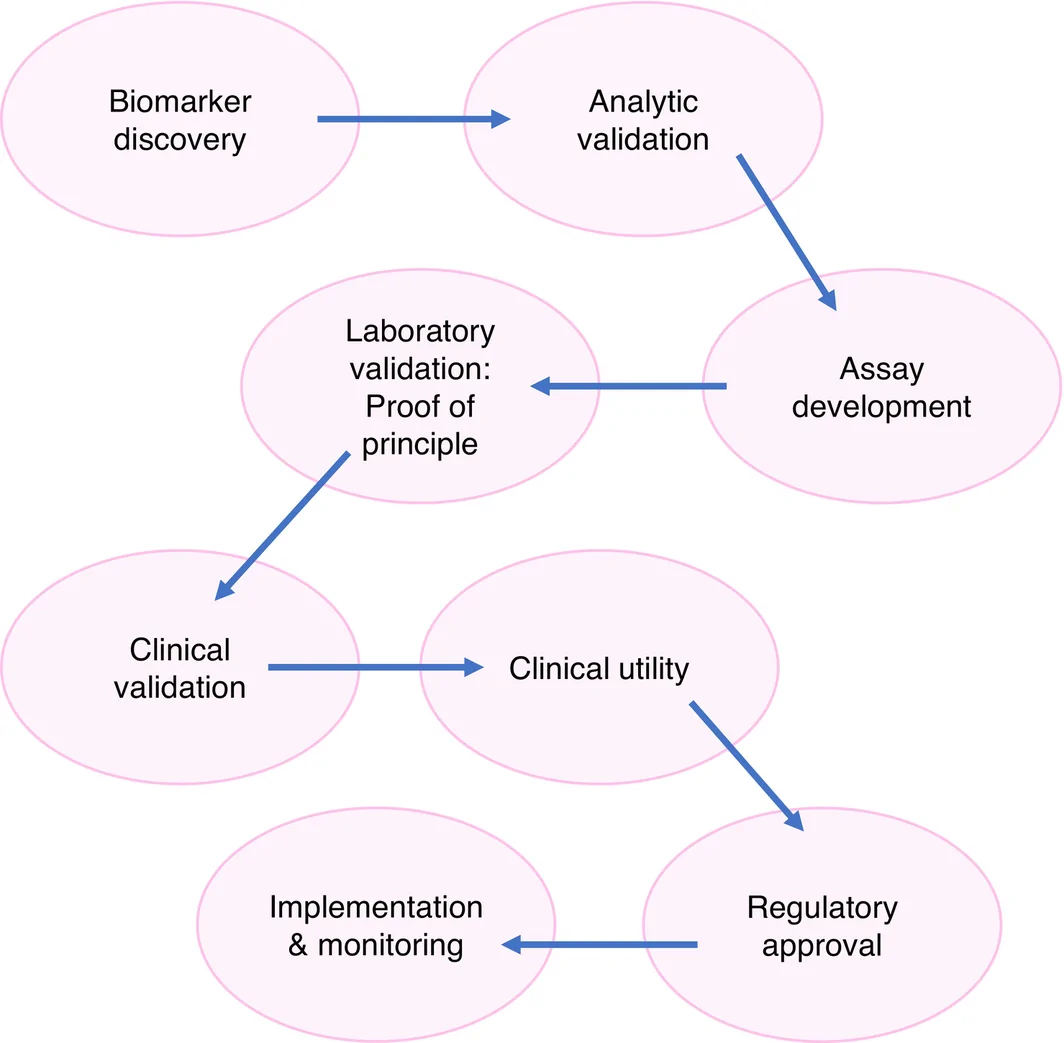
Translational roadmap for periodontitis and peri‐implantitis biomarkers from discovery to implementation.

## 5 Future: Predictive, Personalised and Integrated Periodontal Diagnostics

The future of periodontal diagnostics is expected to move beyond retrospective documentation of tissue loss toward a ‘predictive, preventive and personalised model of care’. Rather than identifying disease only after irreversible destruction has occurred, emerging diagnostic strategies aim to detect individuals and sites at risk ‘before clinical breakdown becomes evident’ [20, 72].

This evolving paradigm will likely involve the integration of ‘validated panels of GCF biomarkers’ with conventional clinical parameters and patient‐level risk factors such as smoking status, glycaemic control and systemic health conditions [20, 50, 72]. Advances in proteomics, metabolomics, and microbiome research are expected to refine biomarker discovery, while technological progress will facilitate the translation of complex molecular data into practical, cost‐effective point‐of‐care assays [73, 74, 75, 76].

Artificial intelligence and machine learning tools are poised to further enhance periodontal diagnostics by integrating multidimensional datasets—including clinical findings, biomarker profiles, behavioural factors, and systemic indicators—to improve diagnostic accuracy and prognostic prediction [50]. A major current role for AI systems currently will be in the rapid and efficient analysis of 2D radiographic images as discussed in detail in the recent EWP review paper [77]. Such systems will assist clinicians in identifying active disease phases, stratifying progression risk, tailoring treatment strategies, and optimising recall intervals on an individual basis.

Ultimately, periodontal diagnostics are likely to evolve from a ‘clinician‐dependent, damage‐based framework’ to a ‘dynamic, biologically informed and patient‐centred system’. Gingival crevicular fluid biomarkers—particularly MMP‐8 and complementary analyte panels—are expected to play a central role in this transformation, supporting earlier diagnosis, personalised treatment planning, and improved long‐term periodontal outcomes [20].

Rakic et al. [49], performed a meta‐analysis on the diagnostic utility of biomarkers in periodontal diagnosis as a contribution to the recent European Periodontal Workshop on diagnosis. The stated aims were to assess host markers with diagnostic accuracy that could be implemented clinically to detect periodontal disease and potentially predict future disease or resolution. The studies had to be sufficiently large and include assessment of diagnostic accuracy and AUC. Of these MMP‐8 was the most reported for both saliva and GCF. When tasking salivary MMPs and cytokines to discriminate between gingivitis and periodontitis they performed poorly (AUC 0.77 and 0.86 respectively—both considered insufficient according to Bossuyt et al. [78]) compared to bone markers (RANKL and calprotectin) (AUC 0.91). This is hardly surprising given the fact that gingivitis and periodontitis are both chronic inflammatory lesions issuing copious inflammatory, transudative and exudative markers into the saliva, whereas bone markers are rooted in the very substance, bone, that is the major tissue lost which differentiates gingivitis and periodontitis. Unfortunately, the majority of studies currently available lack adequate clinical and validation standards [78]. It is also worth emphasising that the index test, i.e., the gold standard against which we need to compare our biomarker tests against, must be true gold‐standard clinical evaluations and diagnoses [12, 78].

When we look to the future of biomarkers, we need to first address some inconvenient truths. The first is that a foundation of choosing the best candidate biomarker should be that we fully understand the pathogenesis of the disease in question. In particular, what differentiates tissue‐contained inflammation (gingivitis or peri‐implant mucositis) from bone destructive inflammation (periodontitis or peri‐implantitis). One of the co‐authors has spent a career attempting to elucidate immunological [79], inflammatory [80], and innate [81] elements coupled with microbial challenge experiments [82], without definitive success either in identifying genetic susceptibility or the elusive pathogenic difference between gingivitis and periodontitis. It may be that as Rakic et al. describe, the best differentiator is one of the actual bone destructions itself, thus somewhat limiting our ability to predict future disease and instead reporting on past disease [49].

A second inescapable reality is that gingivitis and periodontitis are both markedly inflammatory diseases, and similar molecules such as MMPs and cytokines are abundant in both conditions and that MMPs are also critical in remodelling and healing, further questioning their potential utility. A further issue is that we need to be clear about whether any test will be designed to predict, diagnose, monitor, provide prognosis, stage severity, screen or be used to screen or survey populations. The source of the sample, saliva or GCF for example, will also need to be considered in terms of practicality for both study and general utility.

In addition, the only point in performing a clinical diagnostic procedure is if the management of the patient will change based on the outcome of the test. In this regard, much of what we are doing in omics research, non‐hypothesis‐driven and even hypothesis‐driven research is basic research, which is useful as much for understanding the aetiology of disease and only secondarily for developing a diagnostic test. We see omics approaches such as genomics being of more obvious and immediate potential use in such oral conditions as oral squamous cell carcinoma, where recent interest has developed in the use of liquid biopsy techniques to screen for and diagnose this condition [83] but also to understand through genomic and transcriptomics unique elements of the cancer such that therapeutics can be targeted and indeed the omic signature used in follow‐up surveillance.

The recent EWP workshop also included a review of the use of genetics in periodontal diagnosis [84]. While supporting the potential of genome‐wide association studies (GWAS) to determine likelihood of, or susceptibility to, early‐onset forms of periodontal disease, it was considered unlikely to be as useful in the common chronic form of periodontitis, which encompasses multiple factors and has a complex inheritance pattern not conducive to genetic assessment of risk of development. Genetic tests could feasibly uncover non‐changing susceptibility, which might be integrated with environmental and other factors through AI assisted modelling to derive an assessment of how prone an individual might be to periodontitis and thus indicate the need for more frequent and early screening for periodontal disease.

Lastly, we must use resources wisely by conducting studies that meet the criteria checklist but more specifically are adequate in terms of size, comparative indices or gold standards and reported diagnostic validation parameters. We should be aware of the considerable complexity in moving from a hypothesis‐driven or a non‐hypothesis‐driven (−omics) finding that appears to be central to the aetiology of periodontitis, that is, a factor that can separate gingivitis or peri‐implant mucositis, from periodontitis or peri‐implantitis. Apart from critical and highly successful proof‐of‐principle studies, we then need full‐scale, large clinical studies of the test in actual use, focusing on a primary outcome of say differentiation of gingivitis and periodontitis prospectively, and then the further critical statistical assessment of diagnostic accuracy and AUC of the test and just as important and fundamental, is the test practical in the normal clinical workflow, does it add anything in terms of changing clinical decision making. Changes in clinical therapy are more likely to be along the lines of more aggressive therapy, antimicrobial or other pharmacologicall intervention, and more frequent follow‐up and intensive maintenance. At the end of the day the test has to be useful, time‐efficient, and cost‐effective, otherwise it will fail financially and will not remain available. Despite failure as a commercial venture, the knowledge gained will add to our understanding of the aetiopathogenesis and thus be seen to be scientifically valuable. However, commercial gambles are not often undertaken and should not be without a full appraisal of the end outcome potential, i.e., will this test be significantly better than the probe and radiographs we currently have as our mainstay for diagnosis.

## 6 Conclusions

Periodontal and peri‐implant diseases are biologically complex and highly individualised inflammatory conditions. Although traditional clinical parameters such as probing depth, bleeding on probing, and radiographic bone loss continue to serve as the diagnostic foundation, they inherently describe *past* destruction rather than *current* activity or *future* risk. Over several decades, biomarker research has substantially deepened our understanding of host–microbial interactions that drive tissue breakdown, revealing a rich landscape of inflammatory mediators, proteolytic enzymes, bone‐turnover regulators, microbial signatures, and emerging molecular candidates based on non‐hypothesis driven research such as microRNAs.

Gingival crevicular fluid and peri‐implant crevicular fluid have proven especially valuable as site‐specific diagnostic matrices, capturing real‐time molecular events at the lesion interface. Among the numerous candidates investigated, active MMP‐8 has been the most commonly researched diagnostic biomarker across both periodontitis and peri‐implantitis, yet its diagnostic accuracy (DA) is only moderate, questioning its utility in rapid point‐of‐care assays. This could be due to the ubiquity of the metalloproteinases in active disease and repair as well as its multiple cellular sources, innate structural and inflammatory cells. Differentiating soft tissue disease (gingivitis and peri‐mucositis) from disease with soft‐ and hard‐tissue destruction (periodontitis and peri‐implantitis) is unsurprisingly achieved by bone related biomarkers (RANKL/OPG). Yet even if POC biomarkers are developed with excellent DA, the diagnostic matrix, whether it be saliva or GCF, needs careful consideration, with saliva being best for screening and general monitoring and GCF for site‐specific investigations. It is feasible that no single molecule can reflect the multifactorial nature of these diseases, and the strongest current evidence supports combinatorial biomarker panels—integrating host‐response mediators, microbial dysbiosis profiles, and clinical parameters—to improve diagnostic precision and risk stratification.

Peri‐implantitis, with its structural, cellular and vascularity differences (not having a periodontal ligament), accelerated progression, distinct immunological profile, and the potential added complexity of titanium particle–associated inflammation, underscores the need for disease‐specific biomarker strategies rather than direct extrapolation from periodontitis. Early studies combining PICF cytokines, RANKL/OPG ratios, APRIL, and microbial signatures demonstrate the potential of personalised diagnostic models that outperform any single clinical or biochemical measure.

Despite these advances, translation into routine practice remains limited. Methodological heterogeneity, a lack of standardised sampling protocols, biological variability, and insufficient longitudinal validation continue to impede progress. The roadmap ahead requires harmonised protocols, robust multicentre studies, clinically validated thresholds, and technologies that are rapid, affordable, and easily integrated into daily workflows.

Ultimately, the future of periodontal and peri‐implant diagnostics lies in biologically informed, multimodal assessment frameworks that complement traditional examination rather than replace it. As molecular technologies mature and AI‐assisted personalised models evolve, biomarker‐based diagnostics have the potential to transform early detection, risk prediction, and treatment monitoring, bringing us closer to precision periodontology and implantology.

## Author Contributions

The authors conceived, researched, and wrote this review article.

## Disclosure

Ai Statement: AI was not used for creative writing or opinions but was used sparingly for review and suggestions on areas where repetition occurred and for grammatical and spelling errors and checking references. In some cases the text was then changed on the basis of the AI derived suggestion. The figures were created using Power point.

## Conflicts of Interest

The authors declare no conflicts of interest.

## Data Availability

Data sharing not applicable to this article as no datasets were generated or analysed during the current study.

## References

[1] P. N. Papapanou , M. Sanz , N. Buduneli , et al., “Periodontitis: Consensus Report of Workgroup 2 of the 2017 World Workshop on the Classification of Periodontal and Peri‐Implant Diseases and Conditions,” Journal of Clinical Periodontology 45 (2018): S162–S170, 10.1111/jcpe.12946. 29926490

[2] N. J. Kassebaum , E. Bernabé , M. Dahiya , B. Bhandari , C. J. L. Murray , and W. Marcenes , “Global Burden of Severe Periodontitis in 1990–2010: A Systematic Review and Meta‐Regression,” Journal of Dental Research 93 (2014): 1045–1053, 10.1177/0022034514552491. PMC4293771 25261053

[3] G. G. Nascimento , S. Alves‐Costa , and M. Romandini , “Burden of Severe Periodontitis and Edentulism in 2021, With Projections up to 2050: The Global Burden of Disease 2021 Study,” Journal of Periodontal Research 59 (2024): 823–867, 10.1111/jre.13337. 39192495

[4] T. Berglundh , G. Armitage , M. G. Araujo , et al., “Peri‐Implant Diseases and Conditions: Consensus Report of Workgroup 4 of the 2017 World Workshop on the Classification of Periodontal and Peri‐Implant Diseases and Conditions,” Journal of Clinical Periodontology 45 (2018): S286–S291, 10.1111/jcpe.12957. 29926491

[5] G. A. Kotsakis and D. G. Olmedo , “Peri‐Implantitis Is Not Periodontitis: Scientific Discoveries Shed Light on Microbiome‐Biomaterial Interactions That May Determine Disease Phenotype,” Periodontology 2000 86 (2021): 231–240, 10.1111/prd.12372. 33690947

[6] T. Fretwurst , K. Nelson , D. P. Tarnow , H. L. Wang , and W. V. Giannobile , “Is Metal Particle Release Associated With Peri‐Implant Bone Destruction? An Emerging Concept,” Journal of Dental Research 97, no. 3 (2018): 259–265, 10.1177/0022034517740560. 29130804

[7] G. A. Kotsakis and S. M. Ganesan , “Microbial Dysbiosis, Titanium Release, and Peri‐Implantitis,” Journal of Dental Research 104 (2025): 473–480, 10.1177/00220345241307939. PMC12310141 39953673

[8] M. Rakic , L. Canullo , S. Radovanovic , et al., “Diagnostic Value of VEGF in Peri‐Implantitis and Its Correlation With Titanium Particles: A Controlled Clinical Study,” Dental Materials 40, no. 1 (2024): 28–36, 10.1016/j.dental.2023.10.003. 37865576

[9] K. Karlsson , J. Derks , J. L. Wennström , M. Petzold , and T. Berglundh , “Health Economic Aspects of Implant‐Orted Restorative Therapy,” Clinical Oral Implants Research 33, no. 2 (2022): 221–230, 10.1111/clr.13885. 34888933

[10] L. J. A. Heitz‐Mayfield , “Conventional Diagnostic Criteria for Periodontal Diseases (Plaque‐Induced Gingivitis and Periodontitis),” Periodontology 2000 94 (2024): 10–19, 10.1111/prd.12579. 38831568

[11] J. H. Fu and H. L. Wang , “Breaking the Wave of Peri‐Implantitis,” Periodontology 2000 84 (2020): 145–160, 10.1111/prd.12335. 32844418

[12] D. Herrera , M. S. Tonetti , I. Chapple , et al., “Consensus Report of the 20th European Workshop on Periodontology: Contemporary and Emerging Technologies in Periodontal Diagnosis,” Journal of Clinical Periodontology 52, no. S29 (2025): 4–33, 10.1111/jcpe.14152. PMC12286652 40320757

[13] U. Van Der Velden , “Purpose and Problems of Periodontal Disease Classification,” Periodontology 2000 39 (2005): 13–21, 10.1111/j.1600-0757.2005.00127.x. 16135060

[14] X. Xiang , M. G. Sowa , A. M. Iacopino , et al., “An Update on Novel Non‐Invasive Approaches for Periodontal Diagnosis,” Journal of Periodontology 81, no. 2 (2010): 186–198, 10.1902/jop.2009.090419. 20151796

[15] M. S. Gilthorpe , A. T. Zamzuri , G. S. Griffiths , I. H. Maddick , K. A. Eaton , and N. W. Johnson , “Unification of the “Burst” and “Linear” Theories of Periodontal Disease Progression: A Multilevel Manifestation of the Same Phenomenon,” Journal of Dental Research 82, no. 3 (2003): 200–2005, 10.1177/154405910308200310. 12598549

[16] N. P. Lang , R. Adier , A. Joss , and S. Nyman , “Absence of Bleeding on Probing an Indicator of Periodontal Stability,” Journal of Clinical Periodontology 17 (1990): 714–721, 10.1111/j.1600-051X.1990.tb01059.x. 2262585

[17] D. F. Kinane and P. Mark Bartold , “Clinical Relevance of the Host Responses of Periodontitis,” Periodontology 2000 43 (2007): 278–293. 10.1111/j.1600-0757.2006.00169.x 17214845

[18] P. Mark Bartold and T. E. Van Dyke , “Periodontitis: A Host‐Mediated Disruption of Microbial Homeostasis. Unlearning Learned Concepts,” Periodontology 2000 62 (2013): 203–217, 10.1111/j.1600-0757.2012.00450.x. PMC3692012 23574467

[19] P. Mark Bartold and T. E. Van Dyke , “Host Modulation: Controlling the Inflammation to Control the Infection,” Periodontology 2000 75 (2017): 317–329, 10.1111/prd.12169. 28758299

[20] N. Buduneli and D. F. Kinane , “Host‐Derived Diagnostic Markers Related to Soft Tissue Destruction and Bone Degradation in Periodontitis,” Journal of Clinical Periodontology 38 (2011): 85–105, 10.1111/j.1600-051X.2010.01670.x. 21323706

[21] U. K. Gürsoy and A. Kantarci , “Molecular Biomarker Research in Periodontology: A Roadmap for Translation of Science to Clinical Assay Validation,” Journal of Clinical Periodontology 49, no. 6 (2022): 556–561, 10.1111/jcpe.13617. PMC9321848 35322451

[22] D. N. Cagney , J. Sul , R. Y. Huang , K. L. Ligon , P. Y. Wen , and B. M. Alexander , “The FDA NIH Biomarkers, EndpointS, and Other Tools (BEST) Resource in Neuro‐Oncology,” Neuro‐Oncology 20, no. 9 (2018): 1162–1172, 10.1093/neuonc/nox242. PMC6071649 29294069

[23] J. S. Kinney , T. Morelli , T. Braun , et al., “Saliva/Pathogen Biomarker Signatures and Periodontal Disease Progression,” Journal of Dental Research 90, no. 6 (2011): 752–758, 10.1177/0022034511399908. PMC3144122 21406610

[24] W. V. Giannobile , “Salivary Diagnostics for Periodontal Diseases,” Journal of the American Dental Association 143 (2012): 6S–11S, 10.14219/jada.archive.2012.0341. 23024320

[25] L. Steigmann , S. Maekawa , C. Sima , S. Travan , C. W. Wang , and W. V. Giannobile , “Biosensor and Lab‐On‐a‐Chip Biomarker‐Identifying Technologies for Oral and Periodontal Diseases,” Frontiers in Pharmacology 11 (2020): 588480, 10.3389/fphar.2020.588480. PMC7748088 33343358

[26] J. L. Ebersole , H. Hasturk , M. Huber , et al., “Realizing the Clinical Utility of Saliva for Monitoring Oral Diseases,” Periodontology 2000 94 (2024): 203–219, 10.1111/prd.12581. 39010260

[27] I. B. Lamster and J. T. Grbic , “Diagnosis of Periodontal Disease Based on Analysis of the Host Response,” Periodontology 2000 7, no. 1 (1995): 83–99, 10.1111/j.1600-0757.1995.tb00038.x. 9567932

[28] N. Brill and H. Bjorn , “Passage of Tissue Fluid Into Human Gingival Pockets,” Acta Odontologica Scandinavica 17, no. 1 (1959): 11–21.

[29] N. Buduneli , B. Bıyıkoğlu , and D. F. Kinane , “Utility of Gingival Crevicular Fluid Components for Periodontal Diagnosis,” Periodontology 2000 94 (2024): 156–175, 10.1111/prd.12595. 39004819

[30] S. P. Barros , R. Williams , S. Offenbacher , and T. Morelli , “Gingival Crevicular Fluid as a Source of Biomarkers for Periodontitis,” Periodontology 2000 70 (2016): 53–64, 10.1111/prd.12107. PMC4911175 26662482

[31] P. Apse , R. P. Ellen , C. M. Overall , and G. A. Zarb , “Microbiota and Crevicular Fluid Collagenase Activity in the Osseointegrated Dental Implant Sulcus: A Comparison of Sites in Edentulous and Partially Edentulous Patients,” Journal of Periodontal Research 24 (1989): 96–105, 10.1111/j.1600-0765.1989.tb00863.x. 2542514

[32] G. Cimasoni , “The Crevicular Fluid,” Monographs in Oral Science 3 (1974): 1–122. 4600951

[33] G. Cimasoni , Crevicular Fluid Updated (Karger Medical and Scientific Publishers, 1983).

[34] K.’. DF , C.’. CF , J.’. FA , and E' CW , “Neutrophil Chemotactic Behaviour in Patients With Early‐Onset Forms of Periodontitis (I). Leading Front Analysis in Boyden Chambers,” Journal of Clinical Periodontology 16, no. 4 (1989): 242–246, 10.1111/j.1600-051x.1989.tb01648.x. 2715362

[35] E. Adonogjanaki , J. Mooney , D. F. Kinane , and M. J. AE , “The Ability of Gingival Crevicuiar Fluid Acute Phase Proteins to Distinguish Healthy, Gingivitis and Periodontitis Sites,” Journal of Clinical Periodontology 19 (1992): 98–102. 10.1111/j.1600-051x.1992.tb00447.x 1376333

[36] J. Mooney , E. Adonogianaki , and D. F. Kinane , “Relative Avidity of Serum Antibodies to Putative Periodontopathogens in Periodontal Disease,” Journal of Periodontal Research 28 (1993): 444–450. 8254462

[37] B. G. Loos and S. Tjoa , “Host‐Derived Diagnostic Markers for Periodontitis: Do They Exist in Gingival Crevice Fluid?,” Periodontology 2000 39 (2005): 53–72, 10.1111/j.1600-0757.2005.00129.x. 16135064

[38] N. L. Zainal Abidin , N. Latib , F. H. Al‐Bayaty , and M. F. H. Hidayat , “Let‐7e as Potential Diagnostic Biomarkers in Early Detection of Peri‐Implant Diseases: A Cross‐Sectional Study,” Cllinical and Experimental Dental Research 11, no. 3 (2025): e70044, 10.1002/cre2.70044. PMC12051839 40323732

[39] N. A. Ghallab , “Diagnostic Potential and Future Directions of Biomarkers in Gingival Crevicular Fluid and Saliva of Periodontal Diseases: Review of the Current Evidence,” Archives of Oral Biology 87 (2018): 115–124, 10.1016/j.archoralbio.2017.12.022. 29288920

[40] T. Sorsa , U. K. Gursoy , S. Nwhator , et al., “Analysis of Matrix Metalloproteinases, Especially MMP‐8, in Gingival Creviclular Fluid, Mouthrinse and Saliva for Monitoring Periodontal Diseases,” Periodontology 2000 70 (2016): 142–163, 10.1111/prd.12101. 26662488

[41] I. Luchian , A. Goriuc , D. Sandu , and M. Covasa , “The Role of Matrix Metalloproteinases (MMP‐8, MMP‐9, MMP‐13) in Periodontal and Peri‐Implant Pathological Processes,” International Journal of Molecular Sciences 23 (2022): 1806, 10.3390/ijms23031806. PMC8837018 35163727

[42] H. Lähteenmäki , T. Tervahartiala , I. T. Räisänen , et al., “Active MMP‐8 Point‐Of‐Care (PoC)/chairside Enzyme‐Test as an Adjunctive Tool for Early and Real‐Time Diagnosis of Peri‐Implantitis,” Cllinical and Experimental Dental Research 8, no. 2 (2022): 485–496, 10.1002/cre2.537. PMC9033547 35118828

[43] N. Arias‐Bujanda , A. Regueira‐Iglesias , C. Balsa‐Castro , L. Nibali , N. Donos , and I. Tomás , “Accuracy of Single Molecular Biomarkers in Gingival Crevicular Fluid for the Diagnosis of Periodontitis: A Systematic Review and Meta‐Analysis,” Journal of Clinical Periodontology 46 (2019): 1166–1182, 10.1111/jcpe.13188. 31444912

[44] T. Sorsa , S. Alassiri , A. Grigoriadis , et al., “Active MMP‐8 (AMMP‐8) as a Grading and Staging Biomarker in the Periodontitis Classification,” Diagnostics 10, no. 2 (2020): 61, 10.3390/diagnostics10020061. PMC7168924 31979091

[45] S. Gupta , V. Sahni , I. T. Räisänen , et al., “Linking Oral Microbial Proteolysis to aMMP‐8 PoC Diagnostics Along With the Stage and Grade of Periodontitis: A Cross‐Sectional Study,” Oral Diseases 29, no. 1 (2023): 285–289, 10.1111/odi.14008. 34402146

[46] K. Deng , G. Pelekos , L. Jin , and M. S. Tonetti , “Diagnostic Accuracy of a Point‐Of‐Care aMMP‐8 Test in the Discrimination of Periodontal Health and Disease,” Journal of Clinical Periodontology 48, no. 8 (2021): 1051–1065, 10.1111/jcpe.13485. PMC8362205 33998040

[47] T. Sorsa , A. Grigoriadis , D. Sakellari , et al., “On the Accuracy, Sensitivity, and Grading of Mouthrinse Active Matrix Metalloproteinase‐8 (aMMP‐8) Point‐Of‐Care Testing (POCT),” Journal of Clinical Periodontology 48 (2021): 1495–1498, 10.1111/jcpe.13521. 34312914

[48] V. Ö. Öztürk , G. Emingil , K. Umeizudike , et al., “Evaluation of Active Matrix Metalloproteinase‐8 (aMMP‐8) Chair‐Side Test as a Diagnostic Biomarker in the Staging of Periodontal Diseases,” Archives of Oral Biology 124 (2021): 104955, 10.1016/j.archoralbio.2020.104955. 33556789

[49] M. Rakic , E. Calciolari , M. M. Grant , S. Radovanovic , N. Bostanci , and P. M. Preshaw , “Host Markers of Periodontal Diseases: Meta‐Analysis of Diagnostic Accuracy Studies,” Journal of Clinical Periodontology 52 (2025): 155–181, 10.1111/jcpe.14167. 40251882

[50] K. Deng , F. Zonta , H. Yang , G. Pelekos , and M. S. Tonetti , “Development of a Machine Learning Multiclass Screening Tool for Periodontal Health Status Based on Non‐Clinical Parameters and Salivary Biomarkers,” Journal of Clinical Periodontology 51, no. 12 (2024): 1547–1560, 10.1111/jcpe.13856. PMC11651717 37697491

[51] T. Blanco‐Pintos , A. Regueira‐Iglesias , I. Seijo‐Porto , et al., “Accuracy of Periodontitis Diagnosis Obtained Using Multiple Molecular Biomarkers in Oral Fluids: A Systematic Review and Meta‐Analysis,” Journal of Clinical Periodontology 50 (2023): 1420–1443, 10.1111/jcpe.13854. 37608638

[52] G. Baima , M. Corana , G. Iaderosa , et al., “Metabolomics of Gingival Crevicular Fluid to Identify Biomarkers for Periodontitis: A Systematic Review With Meta‐Analysis,” Journal of Periodontal Research 56 (2021): 633–645, 10.1111/jre.12872. 33710624

[53] N. Bostanci , W. Heywood , K. Mills , M. Parkar , L. Nibali , and N. Donos , “Application of Label‐Free Absolute Quantitative Proteomics in Human Gingival Crevicular Fluid by LC/MSE (Gingival Exudatome),” Journal of Proteome Research 9, no. 5 (2010): 2191–2199, 10.1021/pr900941z. 20205380

[54] A. Torres , M. A. Michea , Á. Végvári , et al., “Proteomic Profile of Human Gingival Crevicular Fluid Reveals Specific Biological and Molecular Processes During Clinical Progression of Periodontitis,” Journal of Periodontal Research 58, no. 5 (2023): 1061–1081, 10.1111/jre.13169. 37522282

[55] R. C. Baliban , D. Sakellari , Z. Li , Y. A. Guzman , B. A. Garcia , and C. A. Floudas , “Discovery of Biomarker Combinations That Predict Periodontal Health or Disease With High Accuracy From GCF Samples Based on High‐Throughput Proteomic Analysis and Mixed‐Integer Linear Optimization,” Journal of Clinical Periodontology 40, no. 2 (2013): 131–139, 10.1111/jcpe.12037. PMC3543478 23190455

[56] H. L. Wang , C. Garaicoa‐Pazmino , A. Collins , H. S. Ong , R. Chudri , and W. V. Giannobile , “Protein Biomarkers and Microbial Profiles in Peri‐Implantitis,” Clinical Oral Implants Research 27, no. 9 (2016): 1129–1136, 10.1111/clr.12708. 26424287

[57] X. Wang , Y. Li , Y. Feng , H. Cheng , and D. Li , “Macrophage Polarization in Aseptic Bone Resorption Around Dental Implants Induced by Ti Particles in a Murine Model,” Journal of Periodontal Research 54, no. 4 (2019): 329–338, 10.1111/jre.12633. 30635919

[58] V. Xanthopoulou , I. T. Räisänen , T. Sorsa , D. Tortopidis , and D. Sakellari , “Diagnostic Value of aMMP‐8 and Azurocidin in Peri‐Implant Sulcular Fluid as Biomarkers of Peri‐Implant Health or Disease,” Cllinical and Experimental Dental Research 10, no. 3 (2024): e883, 10.1002/cre2.883. PMC11163026 38853451

[59] I. Fragkioudakis , L. Batas , I. Vouros , and D. Sakellari , “Diagnostic Accuracy of Active MMP‐8 Point‐Of‐Care Test in Peri‐Implantitis,” European Journal of Dentistry 19, no. 3 (2024): 743–748, 10.1055/s-0044-1793843. PMC12182409 39657936

[60] P. M. Duarte , A. C. De Mendonça , M. B. B. Máximo , V. R. Santos , M. F. Bastos , and F. H. Nociti Júnior , “Differential Cytokine Expressions Affect the Severity of Peri‐Implant Disease,” Clinical Oral Implants Research 20, no. 5 (2009): 514–520, 10.1111/j.1600-0501.2008.01680.x. 19302394

[61] M. Rakic , A. Monje , S. Radovanovic , A. Petkovic‐Curcin , D. Vojvodic , and Z. Tatic , “Is the Personalized Approach the Key to Improve Clinical Diagnosis of Peri‐Implant Conditions? The Role of Bone Markers,” Journal of Periodontology 91, no. 7 (2020): 859–869, 10.1002/JPER.19-0283. 31773730

[62] A. Chaparro , V. Ramírez , M. J. Bendek , et al., “Diagnostic Accuracy of Bone‐Related Biomarkers on Peri‐Implantitis: Potential of A‐Proliferation‐Inducing Ligand,” BMC Oral Health 25, no. 1 (2025): 1240, 10.1186/s12903-025-06578-y. PMC12297803 40713636

[63] X. Wu , X. Chen , W. Mi , T. Wu , Q. Gu , and H. Huang , “MicroRNA Sequence Analysis Identifies microRNAs Associated With Peri‐Implantitis in Dogs,” Bioscience Reports 37, no. 5 (2017): BSR20170768, 10.1042/BSR20170768. PMC5964874 28864780

[64] A. Kröger , C. Hülsmann , S. Fickl , et al., “The Severity of Human Peri‐Implantitis Lesions Correlates With the Level of Submucosal Microbial Dysbiosis,” Journal of Clinical Periodontology 45, no. 12 (2018): 1498–1509, 10.1111/jcpe.13023. 30341964

[65] P. Ghensi , V. Heidrich , D. Bazzani , et al., “Shotgun Metagenomics Identifies in a Cross‐Sectional Setting Improved Plaque Microbiome Biomarkers for Peri‐Implant Diseases,” Journal of Clinical Periodontology 52, no. 7 (2025): 999–1010, 10.1111/jcpe.14121. PMC12176464 40467108

[66] A. A. Joshi , S. P. Szafrański , M. Steglich , et al., “Integrative Microbiome‐ and Metatranscriptome‐Based Analyses Reveal Diagnostic Biomarkers for Peri‐Implantitis,” npj Biofilms and Microbiomes 11 (2025): 175, 10.1038/s41522-025-00807-6. PMC12381052 40858628

[67] S. Renvert , C. Widén , and R. G. Persson , “Cytokine and Microbial Profiles in Relation to the Clinical Outcome Following Treatment of Peri‐Implantitis,” Clinical Oral Implants Research 28, no. 9 (2017): 1127–1132, 10.1111/clr.12927. 27422156

[68] R. Teles , K. Moss , J. S. Preisser , et al., “Patterns of Periodontal Disease Progression Based on Linear Mixed Models of Clinical Attachment Loss,” Journal of Clinical Periodontology 45, no. 1 (2018): 15–25, 10.1111/jcpe.12827. 28985450

[69] N. Arias‐Bujanda , A. Regueira‐Iglesias , C. Balsa‐Castro , L. Nibali , N. Donos , and I. Tomás , “Accuracy of Single Molecular Biomarkers in Saliva for the Diagnosis of Periodontitis: A Systematic Review and Meta‐Analysis,” Journal of Clinical Periodontology 47 (2020): 2–18, 10.1111/jcpe.13202. 31560804

[70] H. Zhu , Z. Fohlerová , J. Pekárek , E. Basova , and P. Neužil , “Recent Advances in Lab‐On‐a‐Chip Technologies for Viral Diagnosis,” Biosensors & Bioelectronics 153 (2020): 112041, 10.1016/j.bios.2020.112041. PMC7126858 31999560

[71] A. F. Stadler , P. D. M. Angst , R. M. Arce , S. C. Gomes , R. V. Oppermann , and C. Susin , “Gingival Crevicular Fluid Levels of Cytokines/Chemokines in Chronic Periodontitis: A Meta‐Analysis,” Journal of Clinical Periodontology 43, no. 9 (2016): 727–745, 10.1111/jcpe.12557. 27027257

[72] M. S. Tonetti , H. Greenwell , and K. S. Kornman , “Staging and Grading of Periodontitis: Framework and Proposal of a New Classification and Case Definition,” Journal of Periodontology 89 (2018): S159–S172, 10.1002/JPER.18-0006. 29926952

[73] K. Bao , G. N. Belibasakis , N. Selevsek , J. Grossmann , and N. Bostanci , “Proteomic Profiling of Host‐Biofilm Interactions in an Oral Infection Model Resembling the Periodontal Pocket,” Scientific Reports 5 (2015): 15999, 10.1038/srep15999. PMC4630604 26525412

[74] N. Bostanci , K. Bao , D. Greenwood , A. Silbereisen , and G. N. Belibasakis , “Periodontal Disease: From the Lenses of Light Microscopy to the Specs of Proteomics and Next‐Generation Sequencing,” in Advances in Clinical Chemistry (Academic Press Inc., 2019). 10.1016/bs.acc.2019.07.006 31655732

[75] N. Bostanci and G. N. Belibasakis , “Gingival Crevicular Fluid and Its Immune Mediators in the Proteomic Era,” Periodontology 2000 76 (2018): 68–84, 10.1111/prd.12154. 29193353

[76] G. S. Griffiths , “Formation, Collection and Significance of Gingival Crevice Fluid,” Periodontology 2000 31 (2003): 32–42, 10.1034/j.1600-0757.2003.03103.x. 12656994

[77] R. Farina , A. Simonelli , L. Trombelli , J. B. Ettmayer , J. L. Schmid , and C. A. Ramseier , “Emerging Applications of Digital Technologies for Periodontal Screening, Diagnosis and Prognosis in the Dental Setting,” Journal of Clinical Periodontology 52 (2025): 211–245, 10.1111/jcpe.14156. 40107262

[78] P. M. Bossuyt , J. B. Reitsma , D. E. Bruns , et al., “STARD 2015: An Updated List of Essential Items for Reporting Diagnostic Accuracy Studies,” BMJ 351 (2015): h5527, 10.1136/bmj.h5527. PMC4623764 26511519

[79] D. F. Kinane , D. F. Lappin , and S. Culshaw , “The Role of Acquired Host Immunity in Periodontal Diseases,” Periodontology 2000 94 (2024): 42–59, 10.1111/prd.12562. 38641953

[80] D. F. Kinane , H. Shiba , and T. C. Hart , “The Genetic Basis of Periodontitis,” Periodontology 2000 39 (2005): 91–117, 10.1111/j.1600-0757.2005.00118.x. 16135066

[81] D. F. Kinane , “P. Gingivalis Interactions With Epithelial Cells,” Frontiers in Bioscience 13, no. 13 (2008): 966–984, 10.2741/2736. 17981604

[82] D. F. Kinane , D. R. Demuth , S. U. Gorr , G. N. Hajishengallis , and M. H. Martin , “Human Variability in Innate Immunity,” Periodontology 2000 45, no. 1 (2007): 14–34, 10.1111/j.1600-0757.2007.00220.x. 17850446

[83] D. F. Kinane , J. Gabert , G. Xynopoulos , and E. Guzeldemir‐Akcakanat , “Strategic Approaches in Oral Squamous Cell Carcinoma Diagnostics Using Liquid Biopsy,” Periodontology 2000 94 (2024): 316–328, 10.1111/prd.12567. PMC11579816 38676371

[84] H. Dommisch , D. Hoedke , E. M.‐C. Lu , et al., “Genetic Biomarkers for Periodontal Diseases: A Systematic Review,” Journal of Clinical Periodontology 52 (2025): 182–210, 10.1111/jcpe.14149. PMC12286649 40197750

[85] J. Lindhe and M. Romandini , “Periodontology: The Past, the Present, the Future,” Journal of Periodontal Research 60 (2025): 1–5, 10.1111/jre.13370. PMC11840452 39663179

